# Estimating Fish Body Weight from Morphological Features Using Some Data Mining Algorithms

**DOI:** 10.3390/ani16193124

**Published:** 2026-10-05

**Authors:** Şenol Çelik, Abdulmojeed Yakubu, Alina Makarenko, Ruslan Kononenko, Iryna Kononenko, Andriy Getya, Mykhailo Matvieiev, Nataliia Hryshchenko, Galia Zamaratskaia

**Affiliations:** 1 Biometry and Genetics Unit, Department of Animal Science, Faculty of Agriculture, Bingöl University, Bingöl 12000, Türkiye; senolcelik@bingol.edu.tr; 2 Department of Animal Science, Centre for Sustainable Agriculture and Rural Development, Faculty of Agriculture, Nasarawa State University, Shabu-Lafia Campus, Lafia 950101, Nigeria; abdulmojyak@gmail.com; 3 Department of Hydrobiology and Ichthyology, Faculty of Animal Science and Water Bioresources, National University of Life and Environmental Sciences of Ukraine, Horikhuvats’kyy Shlyakh 19, 03041 Kyiv, Ukraine; almakarenko@nubip.edu.ua (A.M.); ruslan_kononenko@nubip.edu.ua (R.K.); 4 Department of Aquculture, Faculty of Animal Science and Water Bioresources, National University of Life and Environmental Sciences of Ukraine, Horikhuvats’kyy Shlyakh 19, 03041 Kyiv, Ukraine; iryna_kononenko@nubip.edu.ua; 5 Department of Applied Biology, Animal Breeding and Genetics, Faculty of Animal Science and Water Bioresources, National University of Life and Environmental Sciences of Ukraine, Horikhuvats’kyy Shlyakh 19, 03041 Kyiv, Ukraine; getya@ukr.net; 6 Department of Animal Technology, Faculty of Animal Science and Water Bioresources, National University of Life and Environmental Sciences of Ukraine, Heroiv Oborony St, 12A, 03041 Kyiv, Ukraine; matvieiev_mykhailo@nubip.edu.ua (M.M.); nat.hryshchenko@nubip.edu.ua (N.H.); 7 Department of Molecular Sciences, Swedish University of Agricultural Sciences, P.O. BOX 7015, 750 07 Uppsala, Sweden

**Keywords:** data mining, model performance, morphological measurements, fish

## Abstract

The objective was to compare the performance of various data mining algorithms in estimating the body weight of fish. The effects of age, farm type and body morphological characteristics on the body weight of the hybrid silver carp (*Hypophthalmichthys* spp.) were analyzed using some data mining methods. The body weight of the fish was predicted using different morphological measurements taken during its lifetime and after slaughter. Prediction performances of the Chi-Squared Automatic Interaction Detector (CHAID), Classification and Regression Trees (CART), Artificial Neural Networks (ANN), Random Forest (RF), and Multivariate Adaptive Regression Splines (MARS) algorithms were compared. The prediction capabilities of the fitted models were assessed using model fit statistics. The MARS algorithm was demonstrated to be the most effective model for characterizing body weight. Body length showed the greatest relative importance among all traits measured during the fish’s lifetime. When the body length of fish exceeds 145 mm, the body weight is expected to reach 173 g. The MARS algorithm is the most effective model for predicting the body weight of fish, based on the experimental data. It provides an excellent alternative to existing data mining techniques.

## Simple Summary

Fish body weight is one of the most important indicators of growth, health, and production efficiency in aquaculture. Accurately estimating body weight helps farmers improve feeding, management, and harvesting decisions, but weighing every fish is often difficult, time-consuming, and stressful for the animals. In this study, we investigated whether body weight could be estimated from simple body measurements using several modern computer-based prediction methods. We compared five different approaches to determine which one produced the most accurate results for hybrid silver carp. The study also examined how age, farming system, and body shape influenced body weight. Among all methods tested, the multivariate adaptive regression splines approach provided the most accurate predictions. Body length was identified as the most important measurement for estimating weight, and fish longer than 145 mm were predicted to weigh about 173 g. These findings show that body measurements, combined with advanced data analysis methods, can provide a reliable and practical way to estimate fish weight without frequent weighing. This approach can improve farm management, support better production decisions, reduce handling stress for fish, and contribute to more efficient and sustainable aquaculture.

## 1. Introduction

Body weight is one of the most important traits that is normally measured in fish farms. Its measurement in fish is key to the proper management and monitoring of essential production processes. The indices include growth assessment, adjustment of feed, body composition and the determination of the optimal period of harvest [1,2]. In fish farming, where weighing scales are not readily available, body weight has been estimated from morphological traits such as total body length, fork body length, standard body length, body height, head length, head width, and eye diameter [3,4]. These linear traits are easy to measure on farms and are also useful in species differentiation as well as evaluation of within- and between-stock similarities and differences [3].

That is why various machine learning algorithms are widely used for the prediction of the body weight of different farmed animal species based on their body measurements, such as pigs [5], beef cattle [6], sheep [7], rabbits [8], and poultry [9].

However, predictions of body weight from linear body measurements may be limited when the relationships between morphometric traits and body weight are nonlinear or when interactions among predictors are present. Morphometric measurements are often correlated because they represent different dimensions of the same fish, and such correlations may influence model estimation, variable selection, or the interpretation of predictor effects depending on the modeling approach [10]. Therefore, rather than assuming that correlated predictors are simply a problem to be solved, it is important to consider modeling approaches that can accommodate complex relationships among predictors and body weight. This warrants the use of machine-learning and data-mining algorithms such as the chi-squared automatic interaction detector (CHAID), classification and regression trees (CART), artificial neural networks (ANN), random forest (RF), and multivariate adaptive regression splines (MARS). These approaches have been used for body-weight prediction in fish [11,12], narrow-clawed crayfish [13], and crabs [14], demonstrating their potential as alternatives to conventional linear regression approaches.

In a paper, the application of deep learning models for the development of a computer vision system for measurement of live Nile tilapia morphometric traits and prediction of body weight was presented [15]. In a different study, 122 fish’s weight, fish size, sex, season, station, and recorded ectoparasite characteristics in host fish were assessed using two distinct algorithms (MARS and CHAID). To assess the impacts of M. heteranchorus, U. pictorum recorded in the gills, and D. spathaceum recorded in the eyes, chosen as independent variables, the MARS method was developed for the study [16]. In the study by Hassan et al. (2023) [17], biometric measurements such as total length and body weight of 19 fish families were collected from three different locations in the Setiu Wetland in Terengganu. In the authors’ study, the predictive performance of Multiple Linear, Lasso, and Ridge regression models, as well as Tree-Based Regression methods (namely Decision Tree, Random Forest, and XGBoost models), was analyzed. Another study looked into the best predictability by developing prediction models using machine learning approaches (linear regression, multilayer perceptron, Radial Basis Function network, Radial Basis Function regression, and Support Vector Machine for Regression) on G. holbrooki collected from the Gediz River [18]. Weight was employed as an output parameter in a study that used morphometric features such as total length, standard length, and fork length from 321 Sparus aurata samples, 164 females and 157 males captured from İskenderun Bay. The M5P method, the Linear Regression (LR) algorithm, and the Artificial Neural Network (MLP Multi-Layer Perceptron) were used. The study also indicated that, in order to forecast ANN interactions, compared to traditional models, the nonlinear regression model performs better and is more efficient [19].

The choice of an appropriate algorithm is particularly important because the relationship between fish body weight and morphometric characteristics is not necessarily linear. Body dimensions may interact with one another, and the contribution of individual traits may change across different size ranges [4]. Data-mining methods such as CHAID and CART can identify interpretable splitting points and interactions, whereas artificial neural networks (ANN) can model complex nonlinear relationships. Random forest (RF) can improve predictive stability by combining multiple decision trees, while multivariate adaptive regression splines (MARS) can represent nonlinear relationships through adaptive basis functions and identify potential changes in the relationship between predictors and the response [20,21]. Comparative studies have also demonstrated that the predictive performance of different data-mining algorithms may vary according to the dataset and modeling context [22].

Previous studies have demonstrated that machine learning approaches can effectively model nonlinear relationships between fish body measurements and body weight. More recently, computer vision and deep learning approaches have further expanded the possibilities for automated fish-weight estimation. For example, Wang et al. (2026) [23] proposed a multimodal approach based on a two-stream Transformer and adaptive RGB-D fusion for fish-weight estimation, demonstrating the potential of integrating RGB and depth information for automated prediction. Similarly, image-based approaches have been used to extract body measurements and predict fish body weight without relying exclusively on manually measured morphometric traits. These developments indicate a clear transition from conventional morphometric modeling toward automated and multimodal approaches.

In Ukraine, the hybrid silver carp (*Hypophthalmichthys* spp.) is highly valued commercially for its good taste and acceptability [24]. However, information on the estimation of body weight from morphometric measurements using CHAID, CART, ANN, RF and MARS is scarce in the literature. The purpose of this study, therefore, was to estimate the body weight of hybrid silver carp by taking into account many aspects, including age, farm type, and body weight and morphological traits. Additionally, the study sought to identify the variables that had the greatest impact on body weight using five different algorithms.

## 2. Materials and Methods

### 2.1. Determination of Morphological Characteristics in Fish

#### 2.1.1. Study Area

The study was conducted in spring, summer, and autumn periods from 2017 to 2019 in ponds of 3 different fish farms located in the Kyiv region of Ukraine, in particular, 50.577704, 30.095151, 50.46150605619707, 30.28041133104876, and 49.8122, 30.0672.

#### 2.1.2. Data Collection

The target variable evaluated in this study was total fish body weight (BW, g). Predictor variables were classified into two groups based on measurement characteristics: (1) external linear morphometric features obtainable directly from the intact specimen (e.g., body length [BL], body girth [BG], head length [HEL], and forehead width [FORW]), and (2) internal organ parameters necessitating laboratory dissection (liver weight [LiveW] and heart weight [HW]). Internal organ parameters were included alongside external dimensions to investigate systemic allometric interactions and physiological contributors to overall body weight.

The collection of ichthyological material was carried out during stocking and control catching in ponds. The materials for the study included yearlings (1), yearlings (1+), and 2 years old (2) hybrids of the hybrid *Hypophthalmichthys* (*Hypophthalmichthys* spp.). During the morphological analysis, 330 fishes of the hybrid of different size and weight groups caught from the ponds were processed [25,26]. Each fish contributed one observation to the dataset; thus, the sample size of 330 represents 330 unique individual fish. The fish were not individually identified or measured repeatedly over time, and the study did not involve longitudinal or repeated-measures observations. Sample sizes were equal across all groups (N=110 per farm). The age of fish after catching from the ponds was not determined, as the fish species was obtained and reared at the experimental farms according to the methods of artificial reproduction of carps (*Hypophthalmichthys* spp.).

**Figure 1:**
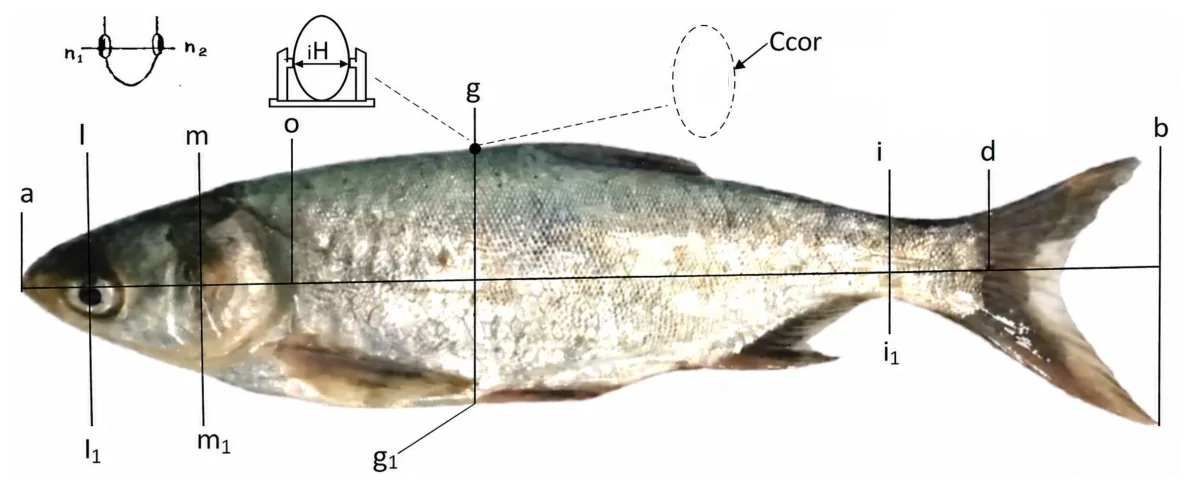
Points for conducting measurements of hybrid *Hypophthalmichthys* (*Hypophthalmichthys* spp.). ab—Body length of whole fish (L); ad—length without caudal fin (standard) (1); od—body length (lcor); gg_1_—the highest height (H); ii_1_—the lowest height (h); ao—head length (1c); n_1_n_2_—forehead width (io); ll_1_—head height through the eye line (hc_1_); mm_1_—head height through the occiput (hc); iH—the highest body thickness was determined using a measuring compass on the line of measurement of the greatest body height; Without fins.

Measurements and determination of fish weight were carried out according to generally accepted methods in ichthyology [25,26]. Measurements were taken according to the accepted methodology [26,27], as shown in Figure 1.

Body measurements were recorded in millimeters, and both body and organ weights were measured in grams.

During the morphometric analysis, the following equipment was used to measure the fish: measuring tapes, rulers and vernier calipers/scale (measuring instrument accuracy: 0.1 mm). Electronic scales (with a maximum capacity of 2.5 kg) were used to determine the fish’s weight.

The biological dataset consisted of 330 hybrid silver carp (*Hypophthalmichthys* spp.) collected from three fish farms located in the Kyiv region of Ukraine during 2017–2019. A total of 110 fish were sampled from each farm. Fish were sampled during the spring, summer, and autumn production periods and represented the available age groups, including yearlings (1), yearlings (1+), and two-year-old fish. Each fish contributed one independent observation to the dataset; therefore, no repeated measurements of the same individual were included.

Fish were selected from the available stocks at the participating farms during the sampling periods. For each sampled fish, body weight and the morphological and biological measurements were recorded. The observed body weight ranged from 2.8 to 444 g.

To provide a transparent description of the sampling structure, the numbers of fish according to farm, year, season, and age group are presented in Table 1.

### 2.2. Statistical Analysis

All statistical analyses were performed using IBM SPSS Statistics version 26 [28] and R software version 4.4.2 [29]. The Artificial Neural Network (ANN) analysis, CHAID and CART algorithms were conducted using SPSS, whereas the remaining machine learning algorithms (RF and MARS) were implemented in R.

To ensure a consistent comparison among the machine-learning models, the dataset was randomly partitioned at the individual-fish level into training (70%, n = 231 (n: number of observations)) and testing (30%, n = 99) subsets. Because each fish contributed only one observation, no individual fish was represented in both subsets. The same training and testing subsets were used for all five algorithms (RF and MARS) to ensure that their predictive performances were evaluated on identical observations. The distributions of farm, age class, year, and season in the training and testing subsets were examined to assess the comparability of the two subsets. No explicit stratification by farm, age class, year, or season was applied; the partition was performed by random sampling at the individual-fish level.

Artificial Neural Networks (ANN): Implemented in IBM SPSS Statistics (v26) using a Multilayer Perceptron (MLP) architecture. The model structure comprised an input layer, one hidden layer, and an output layer. The input layer contained 12 input units (representing farm location dummies [farm = 1, 2, 3], age, BL, THH, BG, HEL, FORW, HH eye, HH occi, LiveW, and HW) plus a bias term. A single hidden layer with 7 neurons (H(1:1) to H(1:7)) was configured based on training dataset optimization. The hyperbolic tangent function was specified as the activation function for the hidden layer, while the identity function was applied to the single output neuron (body weight, BW). Model parameters were optimized using the Scaled Conjugate Gradient (SCG) training algorithm.

CHAID: Implemented in IBM SPSS Statistics. Tree growth was controlled using Pearson’s chi-square test for categorical splits (*p* < 0.05 for splitting and merging) with Bonferroni adjustment. The maximum tree depth was restricted to 3 levels to prevent overfitting, with minimum parent node size set to n = 30 and minimum child node size set to n = 15.

CART: Implemented in IBM SPSS Statistics using the Gini/least-squares deviation splitting criterion for continuous response modeling. The maximum tree depth was capped at 4. The minimum number of cases was set to 30 for parent nodes and 15 for terminal nodes. Pruning was applied based on variance reduction to obtain the most parsimonious tree.

Random Forest (RF): Implemented in R using the randomForest package (R software version 4.4.2). Hyperparameters were tuned over a grid using 10-fold cross-validation. The optimal model comprised ntree=500 trees and mtry=3 (number of variables randomly sampled as candidates at each split, evaluated around $\sqrt{p}\approx 3$). Minimum node size was fixed at 5.

Multivariate Adaptive Regression Splines (MARS): Implemented in R (v4.4.2) using the earth package. Model optimization was conducted using 10-fold cross-validation on the training set (n = 231). To capture multi-trait combined effects, the maximum degree of interaction was set to 3. The forward stepwise function selection considered up to 21 basis functions, which were subsequently pruned based on Generalized Cross-Validation (GCV) optimization, yielding a minimum GCV of 117.0.

The performance of all five models (CHAID, CART, ANN, RF, and MARS) was evaluated using the same independent testing dataset and the same goodness-of-fit and prediction criteria.

#### 2.2.1. The Chi-Squared Automatic Interaction Detector (CHAID) Algorithm

The chi-squared automatic interaction detector (CHAID) algorithm [30] introduced the CHAID algorithm in 1975. A predictive analysis is carried out to determine a criterion variable connected to the remaining variables, the independent ones, using Chi-square Automatic Interaction Detection. The segments are formed by the relationship between the independent variables and the criterion. This enables researchers to use a variety of independent variables (predictors) and the criterion variable to determine the segmentation [31,32].

Similarity between attributes is used as a separator criterion in the CHAID Rule-Based Algorithm, also known as Chi-square automated interaction detection. This algorithm chooses the best feature in each decision node based on the chi-square ($\chi^{2}$) criterion.

#### 2.2.2. Classification and Regression Trees (CART) Algorithm

One of the data exploration strategies, the decision tree methodology, has an algorithm called CART (Classification and Regression Trees). CART is a straightforward yet effective technique. In addition to describing the relationship between the response variable (dependent variable) and one or more predictor variables (independent variables), CART seeks to produce a set of data that accurately identifies a classification. The resulting tree model is dependent on the scale of the response variable; a regression tree is produced if the data response variable is continuous, and a classification tree is produced if the response variable has a categorical scale [33].

Getting an accurate set of data to use as a classifier’s identifier is the primary goal of CART. The three steps of the CART algorithm are as follows [34]: These include creating a classification tree, pruning a classification tree, and figuring out the best classification tree.

#### 2.2.3. Artificial Neural Networks (ANN)

Artificial neural networks (ANNs) are computational models that behave similarly to the human nervous system. The mathematical procedures and a set of parameters needed to determine the output are the basis for the implementation of these kinds of networks [35].

An input layer, an output layer, and one or more hidden layers are only a few of the processing elements, also known as nodes, that make up an ANN’s conventional structure [36]. An activation function is used in an artificial neural network (ANN) [37,38]:

Numerous intricately linked processing units known as neurons make up the system. While the output layer creates the ultimate outcome, the input layer receives the data. Every neuron has an activation function and a threshold value, and every connection has a connection weight [39]. Depending on the sign of the input’s weight, it is determined whether each input has a positive or negative weight [40]. The output is produced using the signal from the activation value, which is the weighted sum of the summing unit [41].

#### 2.2.4. The Random Forest Algorithm

Random Forest (RF) model was constructed from multiple decision trees generated from bootstrap samples of the training data, with a randomly selected subset of predictors considered at each split. The main model parameters, including the number of trees, the number of predictors considered at each split, and the minimum terminal-node size, were specified or tuned using the training dataset only. The final RF model was subsequently applied to the independent testing dataset.

The Random Forest algorithm is a decision tree-based ensemble learning technique [42]. In machine learning, bagging, also known as bootstrap aggregating, is a common method for merging many basic models to create efficient models [43].

#### 2.2.5. Multivariate Adaptive Regression Spline (MARS)

A nonparametric modeling method called MARS augments the linear model with nonlinearities and variable interactions [20]. The following is how the model is expressed:

(1) $$y_{t}=f\left(x_{t}\right)=\;\beta_{0}+\sum_{i=1}^{k}\beta_{i}B\left(x_{it}\right)$$

where $\beta_{i}$, which range from i = 1, …, k, are the model parameters for the corresponding $x_{it}$ variables and $y_{t}$ is the response variable at instant t. The intercept is represented by the value $\overset{ˇ}{0}$, and the basis functions each $B\left(x_{it}\right)$ may be expressed as where c is a threshold value, and k is the number of explanatory variables, which includes interactions of the predictor variables [44].

After identifying the fundamental functions and knots, the functions with the highest estimated performance are determined using the least squares approach [45]. Generalized Cross Validation (GCV) measurement serves as the basis for model selection [46].

For the MARS model, the cross-validated coefficient of determination (CVR^2^) was additionally used to assess predictive performance under cross-validation [47].

#### 2.2.6. Model Evaluation

These goodness-of-fit statistics’ formulations are shown below [48,49,50].

Coefficient of determination:

(2) $$R^{2}\;=\;1-\;\frac{\sum_{i=1}^{n}{\left(y_{i}-\hat{y_{i}}\right)}^{2}}{\sum_{i=1}^{n}{\left(y_{i}-{\bar{y}}_{i}\right)}^{2}}$$

Adjusted coefficient of determination:

(3) $${Adj.R}^{2}\;=\;1\;-\;\frac{\frac{1}{n\;-\;k\;-\;1}\sum_{i=1}^{n}{\left(y_{i}\;-\;\hat{y_{i}}\right)}^{2}}{\frac{1}{n\;-\;1}\sum_{i=1}^{n}{\left(y_{i}\;-\;{\bar{y}}_{i}\right)}^{2}}$$

Root-mean-square error (RMSE) given by the following formula:

(4) $$RMSE\;=\;\sqrt{\frac{\sum_{i=1}^{n}{\left(y_{i}\;-\;\hat{y_{i}}\right)}^{2}}{n}\;\;}$$

Mean absolute error

(5) $$MAE=\frac{\sum_{i=1}^{n}\left|y_{i}-\hat{y_{i}}\right|}{n}$$

The following additional model evaluation criteria were also computed to evaluate model performance [51,52,53].

Mean absolute percentage error (MAPE);

(6) $$MAPE=\frac{1}{n}\sum_{i=1}^{n}\left|\frac{y_{i}-{\hat{y}}_{i}}{y_{i}}\right|*100$$

Mean absolute deviation (MAD);

(7) $$MAD=\frac{1}{n}\sum_{i=1}^{n}\left|y_{i}-{\hat{y}}_{i}\right|$$

Standard deviation ratio (SD_ratio_);

(8) $${SD}_{ratio}=\sqrt{\frac{\frac{1}{n\;-\;1}\sum_{i=1}^{n}{\left(\varepsilon_{i}-\bar{\varepsilon }\right)}^{2}}{\frac{1}{n\;-\;1}\sum_{i=1}^{n}{\left(y_{i}-\bar{y}\right)}^{2}}}$$

Global relative approximation error (RAE);

(9) $$RAE=\sqrt{\frac{\sum_{i=1}^{n}{\left(y_{i}-\hat{y_{i}}\right)}^{2}}{\sum_{i=1}^{n}y_{i}^{2}}}$$

Akaike information criterion (AIC) calculated as:

(10) $$AIC\;=\;n\;ln\left[\frac{1}{n}{\left(y_{i}\;-\;\hat{y_{i}}\right)}^{2}\right]\;+\;2k,\;\;\;if\frac{n}{k}\;>40$$

or:

(11) $${AIC}_{c}\;=\;n\;ln\left[\frac{1}{n}{\left(y_{i}\;-\;\hat{y_{i}}\right)}^{2}\right]\;+\;2k+\frac{2k(k+1)}{n\;-\;k\;-\;1},\;\;\;\;\;\;\;\;\;\;\;\;\;\;\;\;\;otherwise$$

where $y_{i}$: observed value of the response variable, $\hat{y_{i}}$: the predicted value of the response variable, $\bar{y}$: mean of the observed values, $\varepsilon_{i}$: the residual value of the ith value, $\bar{\varepsilon }$: mean of the residual values, *k*: number of independent variables included significantly in the model, and n: total sample size.

The response variable was body weight (BW). The predictor variables were classified according to whether they could be obtained from a live fish or required post-mortem examination. The live-fish morphometric predictors included head length (HEL), forehead width (FORW), head height through the eye line (HH eye), head height through the occiput (HH occi), body length (BL), the highest height (THH), and body girth (BG). These measurements can be obtained directly from a live fish without slaughter and therefore have potential practical application for non-destructive body-weight estimation. In contrast, liver weight (LiveW) and heart weight (HW) were obtained post-mortem.

## 3. Results

**Figure 2:**
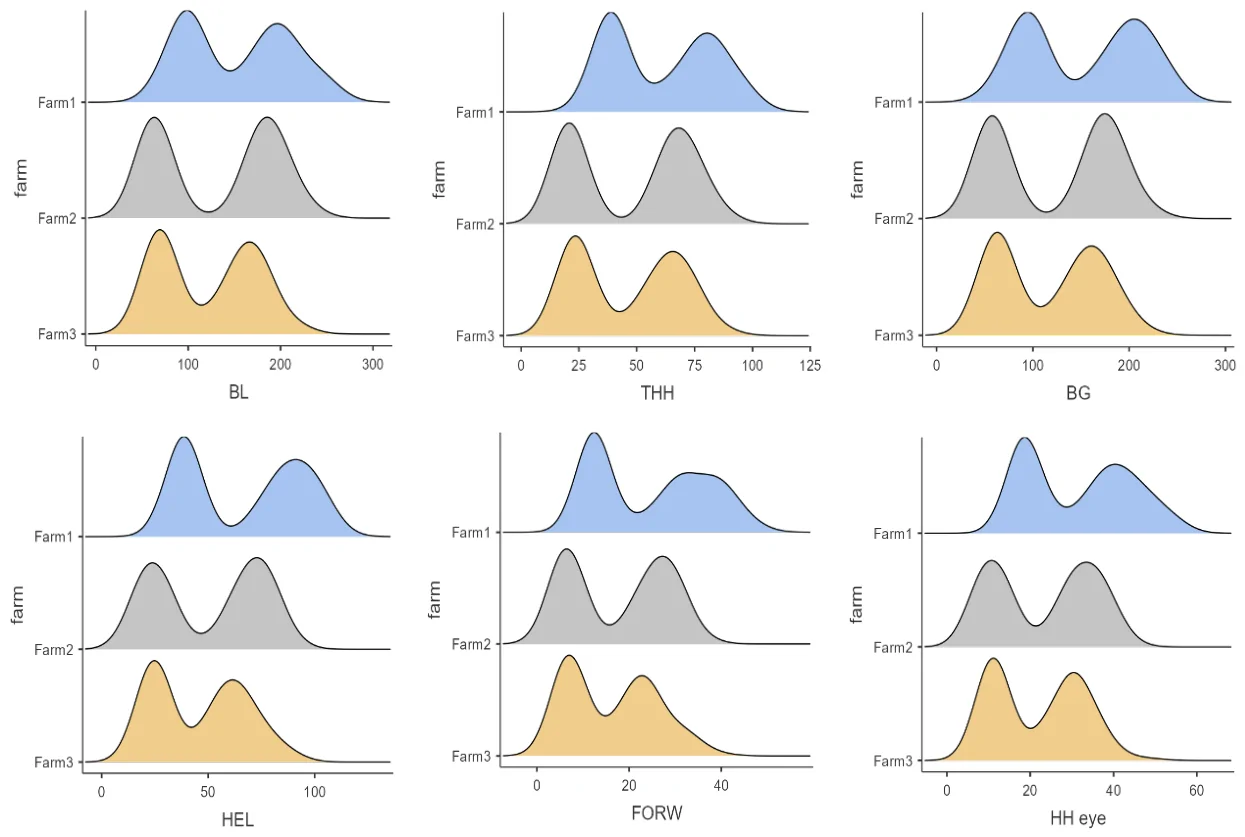

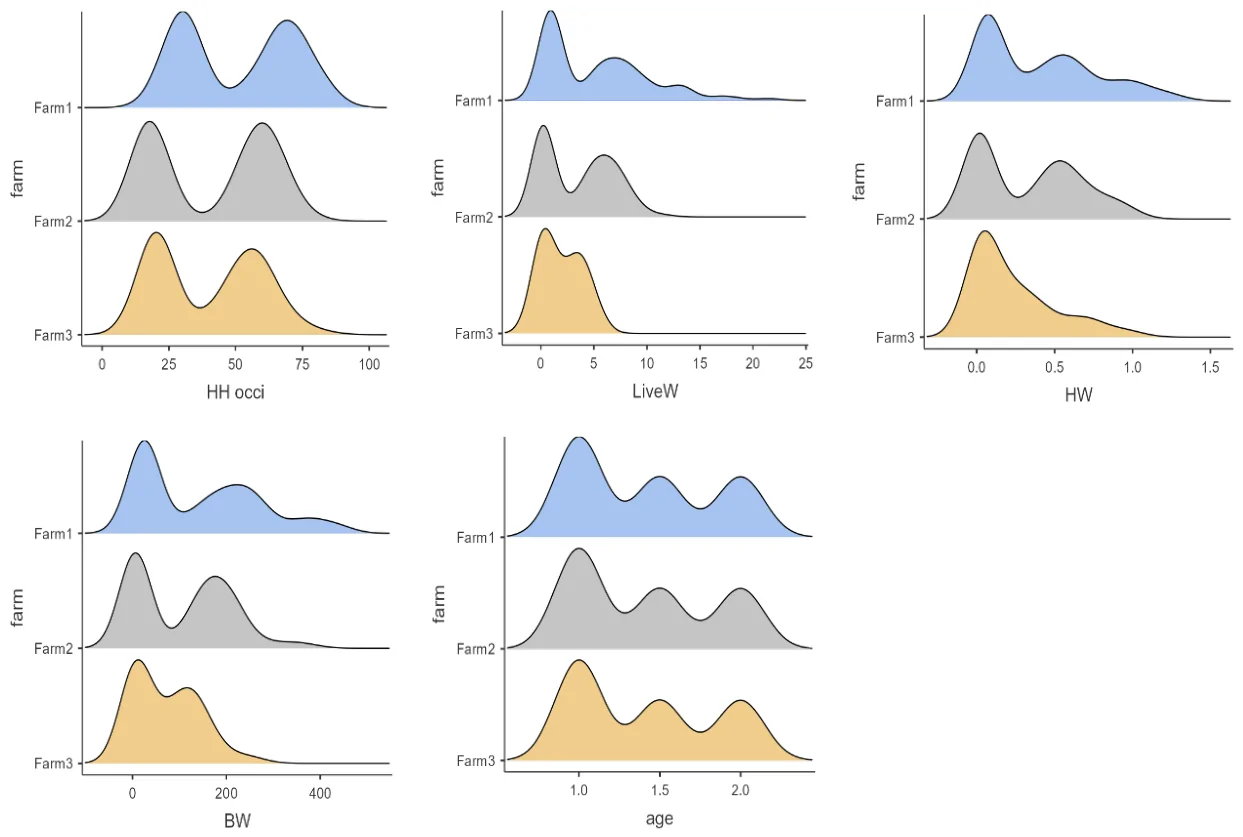
Graphs of body morphologic characteristics of fish.

Descriptive statistics of body morphologic characteristics of fish are given in Table 1 and Figure 2.

The descriptive statistics for the morphometric and body weight characteristics of fish across the three farms are summarized in Table 1.

The age distribution of fish was consistent among the farms, with mean values ranging from 1.927 to 1.969 years.

Specifically, fish reared in Farm1 exhibited substantially higher mean body weight (BW=144.422±127.04 g) compared to those in Farm2 (106.171±99.976 g) and Farm3 (70.497±67.449 g). Similarly, linear body measurements including body length (BL), body girth (BG), and head length (HEL) as well as internal organ weights (LiveW and HW) were markedly greater in Farm1 than in the other two farms. These findings suggest that environmental factors, stocking density, or farm management practices may have influenced growth performance independently of age.

The correlation coefficients between the body morphological characteristics of fish are shown in Table 2.

As shown in Table 2, all investigated body measurements and weight-related traits were positively and highly significantly correlated with each other (*p* < 0.001). The correlation coefficients ranged from 0.825 to 0.993. The strongest association was observed between THH and BG (r = 0.993), followed by THH and HH occi (r = 0.991) and BG and HH occi (r = 0.991). Liver weight (LiveW) was also strongly correlated with the other traits, with correlation coefficients ranging from 0.825 for HW to 0.906 for BW. These findings indicate a strong interdependence among body measurements and weight-related traits.

### 3.1. CHAID Algorithm

**Figure 3:**
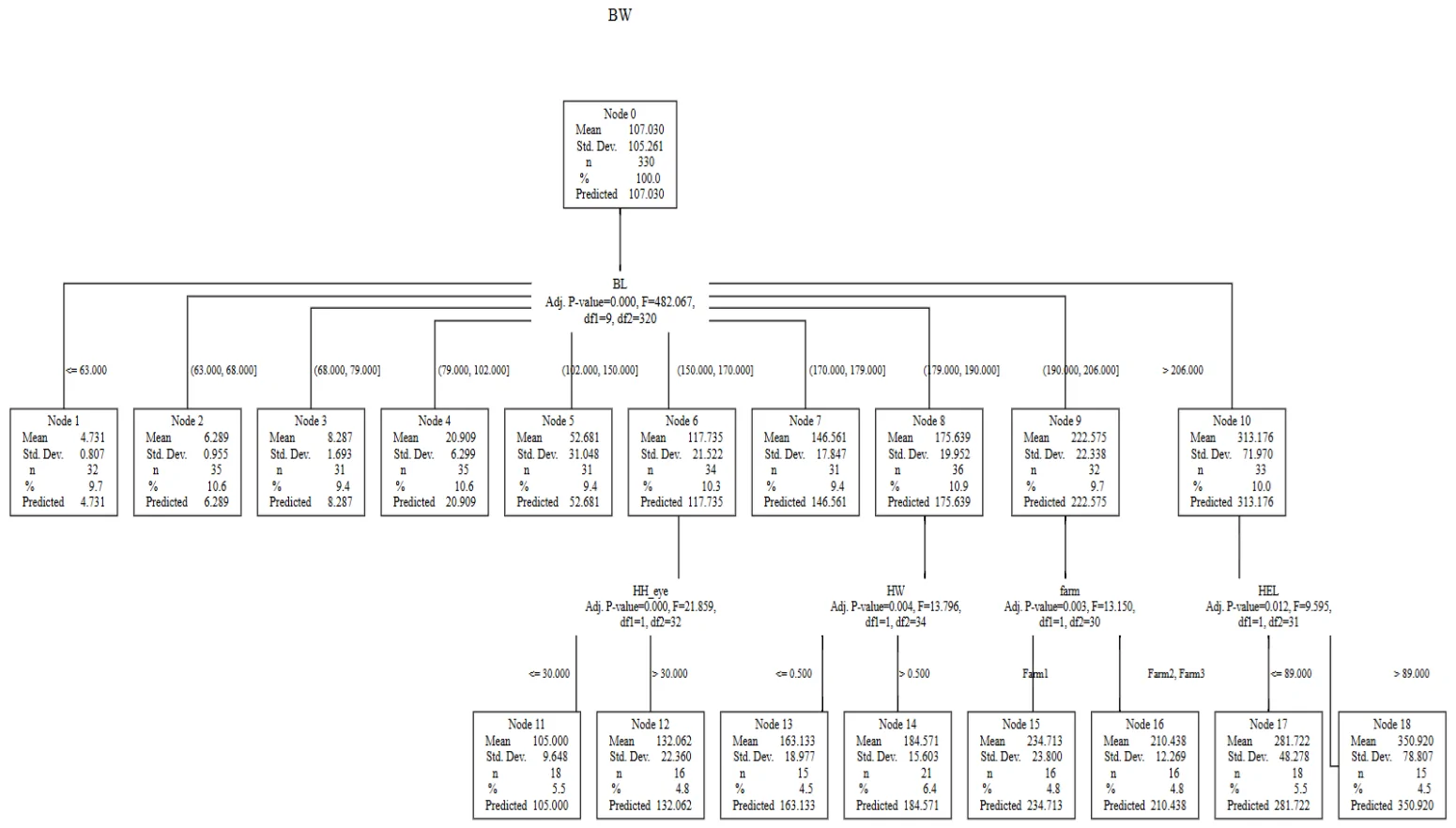
The regression tree diagram constructed by the CHAID tree-based algorithm.

Factors affecting body weight in fish using the CHAID algorithm are displayed in Figure 3.

To evaluate model performance, data partitioning was applied. For decision tree models (CHAID and CART) implemented in IBM SPSS Statistics, tree structures and topological node splits were developed on the complete dataset (N=330) utilizing internal 10-fold cross-validation pruning to preserve total sample depth and optimize biological decision rules. However, to ensure an unbiased benchmarking framework against R-implemented ensemble models (RF and MARS), the predictive accuracy statistics ($R^{2}$, Adj-$R^{2}$, RMSE, MAE, MAPE, and SDratio) for all five algorithms were evaluated on the identical hold-out test validation subset.

**Figure 4:**
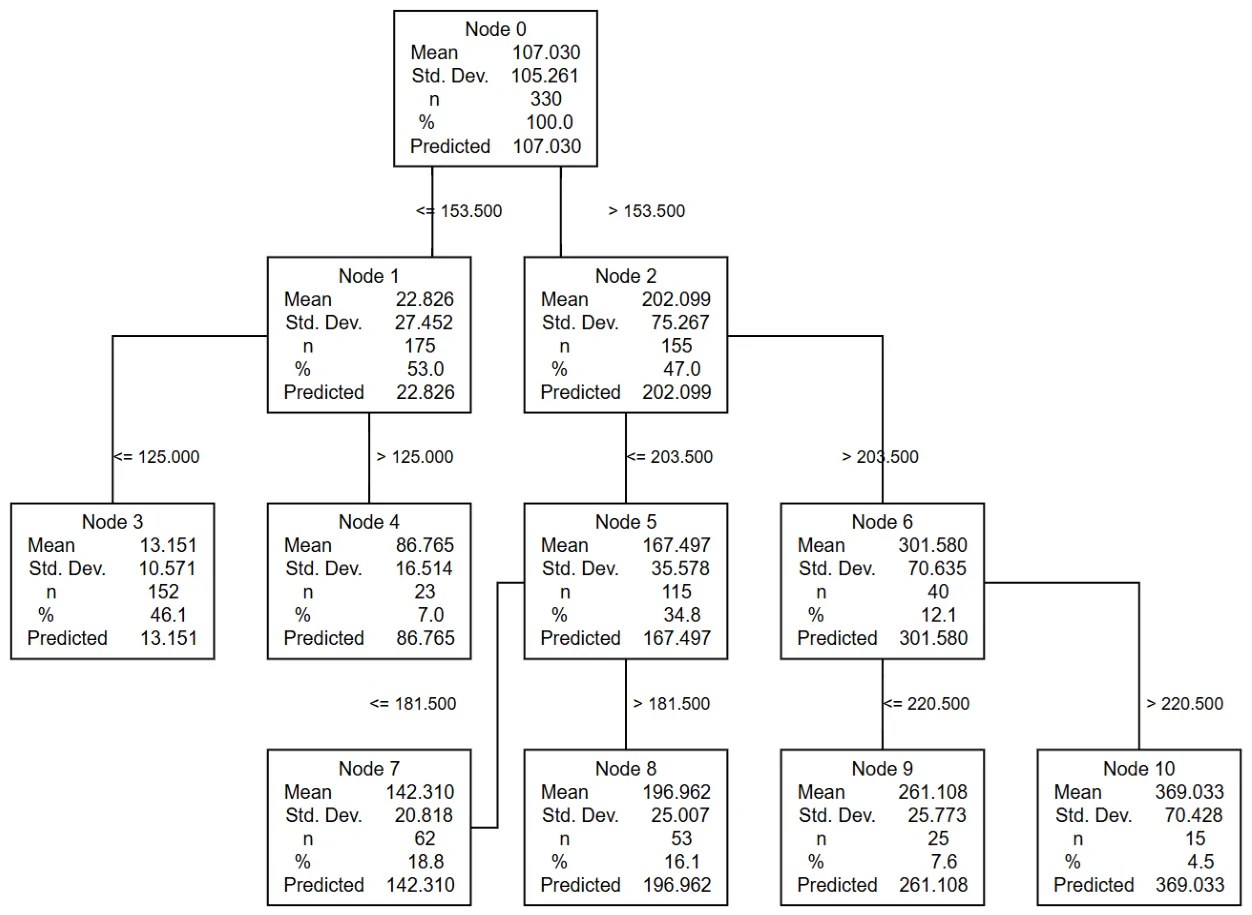
The regression tree diagram constructed by the CART tree-based algorithm.

All 330 fish in the study are included in Node 0 at the top of the regression tree structure, with an overall mean body weight (BW) of 107.030 (S = 105.261) g. Based on the BL trait, Node 0 was divided into ten smaller subgroups (Nodes 1–10) that were accessible at the first tree depth. Among them, terminal nodes with the numbers 1, 2, 3, 4, 5, and 7 were unaffected by any predictor at the second tree depth of the regression tree structure that was built here. The average BW ranged from 4.731 to 313.176 g, increasing progressively from Node 1 through Node 10 as BL increased (Figure 4). Thus, BL and BW showed a positive association at the first tree depth. The average BW of Node 1, a subgroup of the hybrid *Hypophthalmichthys* (*Hypophthalmichthys* spp.) with BL ≤ 63, was 4.731 g. At the second tree depth, head height through the eye line, HW, farm and HEL, respectively, created nodes 11–18. On BW, these four predictors were significant in the second degree. The highest average BW (350.920 g) predicted here was obtained by Node 18 in the regression tree structure. At the second level of the CHAID tree, head height through the eye line, heart weight (HW), farm, and head length (HEL) contributed to subsequent partitioning of the observations. The inclusion of farm as a splitting variable indicates that differences among the sampled farms contributed to the prediction of body weight within the study population. The fact that farm generated a node at the second tree depth indicates that farm membership contained predictive information within the present dataset, but it does not establish a direct causal relationship between farm identity and fish body weight. Importantly, because the environmental parameters underlying the differences among farms were not systematically measured, the model cannot identify which specific conditions were responsible for this effect. Consequently, the predictive contribution of farm should not be assumed to remain stable in an unrepresented production environment. For example, the present model was not independently evaluated on a previously unseen Farm 4, and therefore its performance on a new farm cannot be guaranteed.

### 3.2. CART Algorithm

Factors affecting the body weight of fish using the CART algorithm are presented in Figure 4. In the CART regression tree structure, body girth (BG) was identified as the primary predictor affecting body weight (BW). At the first tree depth, Node 0 was divided into two subgroups according to BG values: Node 1 (BG ≤ 153.5) and Node 2 (BG > 153.5). Fish in Node 2 had a considerably higher mean BW (202.099 g) than those in Node 1 (22.826 g), indicating a strong positive relationship between BG and BW.

Node 1 was further partitioned into Nodes 3 and 4 based on body length (BL ≤ 125 and BL > 125). The mean BW increased from 13.151 g in Node 3 to 86.765 g in Node 4, suggesting that fish with greater BL values tended to have higher BW within the lower BG group.

Similarly, Node 2 was divided into Nodes 5 and 6 according to BL values (BL ≤ 203.5 and BL > 203.5). Fish in Node 6 had a substantially greater mean BW (301.580 g) compared with those in Node 5 (167.497 g). This finding demonstrated that BL remained an important determinant of BW even among fish with higher BG measurements.

At the next tree depth, Node 5 was further split into Nodes 7 and 8 based on BL values (BL ≤ 181.5 and BL > 181.5). The corresponding mean BW values were 142.310 g and 196.962 g, respectively, indicating that BW increased with increasing BL.

Likewise, Node 6 was partitioned into Nodes 9 and 10 according to BL values (BL ≤ 220.5 and BL > 220.5). The highest mean BW (369.033 g) in the entire regression tree structure was observed in Node 10, whereas Node 9 had a lower mean BW of 261.108 g. These results clearly indicated that simultaneous increases in BG and BL contributed substantially to higher BW in fish.

### 3.3. ANN Model

**Figure 5:**
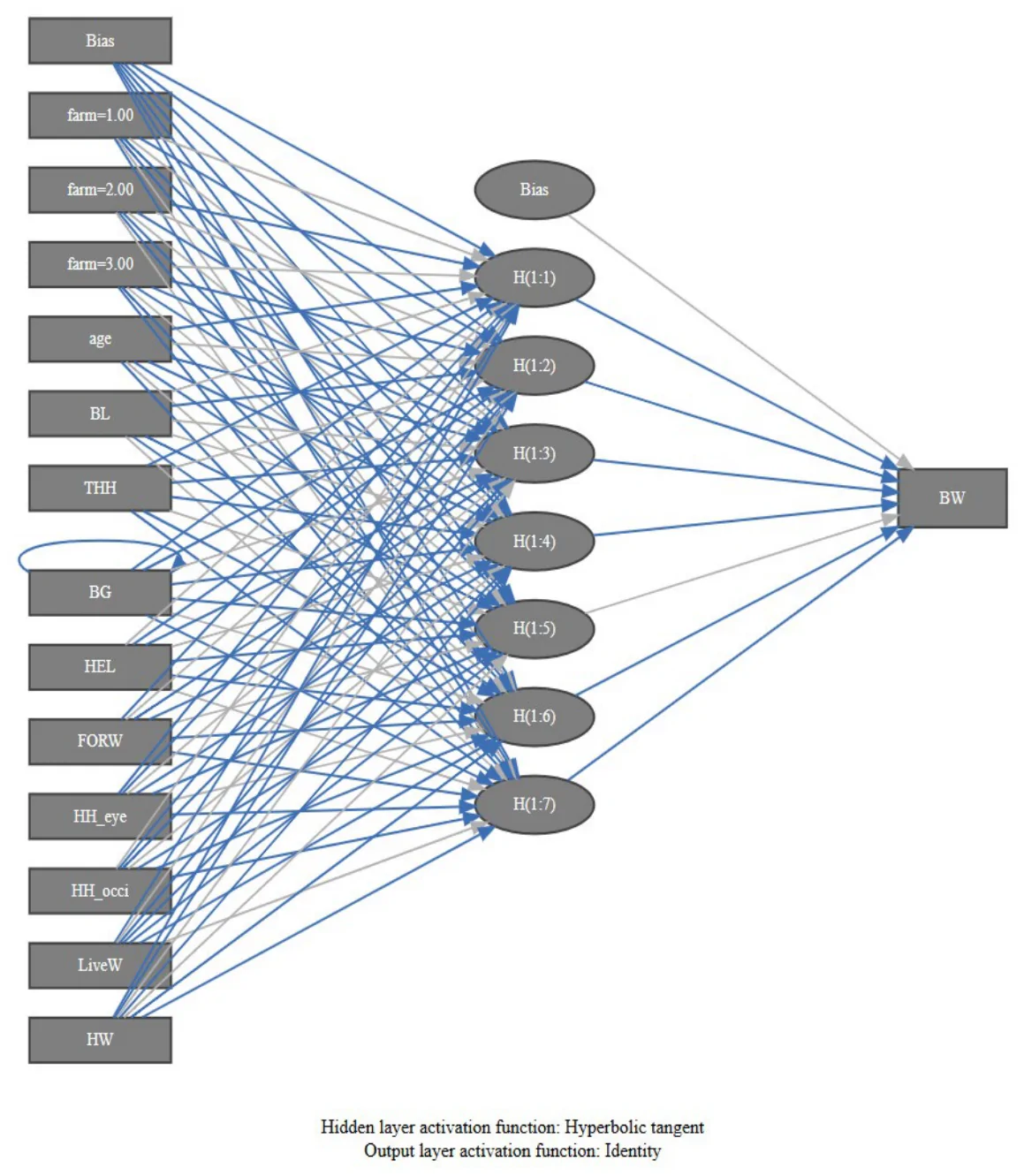
Artificial neural network architecture.

A multilayer perceptron artificial neural network model was developed to assess its suitability for the data. In this study, an ANN with 10-fold cross-validation was used. Figure 5 demonstrates the connections for the ANN.

In Figure 5, the activation function in the hidden layer in artificial neural network architecture as hyperbolic tangent, identity as activation function in the output layer function was applied. Table 3 lists the model’s connection weights and threshold levels.

The predicted BW was found to be expressed as follows:

$${BW}_{nor}=tanh(0.859-0.181*H_{1}-0.334*H_{2}-0.346*H_{3}-0.210*H_{4}-0.181*H_{5}-0.950*H_{6}-1.006*H_{7}$$

$$H_{1}=\frac{1}{1+e^{-x_{1}}},H_{2}=\frac{1}{1+e^{-x_{2}}},H_{3}=\frac{1}{1+e^{-x_{3}}},H_{4}=\frac{1}{1+e^{-x_{4}}}$$

$$H_{5}=\frac{1}{1+e^{-x_{5}}},H_{6}=\frac{1}{1+e^{-x_{6}}},H_{7}=\frac{1}{1+e^{-x_{7}}}$$

$$x_{1}=-0.402+0.348*farm1+0.082*farm2+0.404*farm3-0.509*age-0.026*BL+0.245*THH+0.120*BG-0.400*HEL-0.322*FORW+0.152*HH\;eye-0.378*HH\;occi+0.204*LiveW+0.087*HW$$

$$x_{2}=-0.038-0.398*farm1-0.226*farm2-0.166*farm3+0.412*age+0.178*BL+0.109*THH-0.266*BG+0.007*HEL-0.021*FORW+0.471*HH\;eye-0.235*HH\;occi+0.001*LiveW-0.156*HW$$

$$x_{3}=-0.234+0.341*farm1+0.199*farm2+0.266*farm3-0.528*age-0.275*BL-0.456*THH+0.356*BG-0.461*HEL+0.367*FORW+0.363*HH\;eye+0.405*HH\;occi+0.127*LiveW-0.426*HW$$

$$x_{4}=0.352-0.027*farm1+0.156*farm2+0.405*farm3+0.401*age+0.253*BL-0.113*THH+0.327*BG+0.474*HEL+0.091*FORW+0.291*HH\;eye-0.084*HH\;occi+0.262*LiveW+0.357*HW$$

$$x_{5}=0.103-0.289*farm1+0.223*farm2-0.226*farm3-0.051*age-0.319*BL+0.110*THH+0.258*BG-0.276*HEL+0.070*FORW-0.156*HH\;eye-0.249*HH\;occi+0.305*LiveW-0.295*HW$$

$$x_{6}=0.633+0.458*farm1+0.414*farm2+0.460*farm3-0.012*age-0.305*BL-0.074*THH-0.543*BG-0.080*HEL-0.392*FORW-0.287*HH\;eye+0.030*HH\;occi+0.190*LiveW+0.302*HW$$

$$x_{7}=0.414+0.287*farm1+0.254*farm2+0.233*farm3-0.052*age-0.717*BL-0.185*THH+0.201*BG+0.430*HEL+0.130*FORW+0.043*HH\;eye-0.081*HH\;occi-0.471*LiveW-0.018*HW$$

The learning Sum of Squares Error (SSE) value and relative error in the ANN model were 2.353 and 0.021, respectively. The error of the test (SSE) and relative error was 2.544 and 0.054, respectively. Table 4 displays the percentage importance of the independent variables.

When Table 4 was investigated, among the independent variables considered to be effective on body weight in the output layer, farmer is 0.007, age 0.048, BL 0.358, THH 0.109, HEL 0.067, FORW 0.057, HH eye 0.062, HH occi 0.049, LiveW 0.131 and HW 0.054. BL has the highest and 100% effect on body weight of fish from the terminal in this model. In addition, LiveW is the second most important independent variable with a rate of 36.5%, while THH has an effect of 30.5%. While HEL, HH eye, BG, FORW, HW, and HH occi have effects of 18.6%, 17.4%, 16.5%, 15.9%, 15.1%, 13.8%, and 13.4%, respectively, farm has the least effect, with a rate of 1.8% on body weight from the terminals.

**Figure 6:**
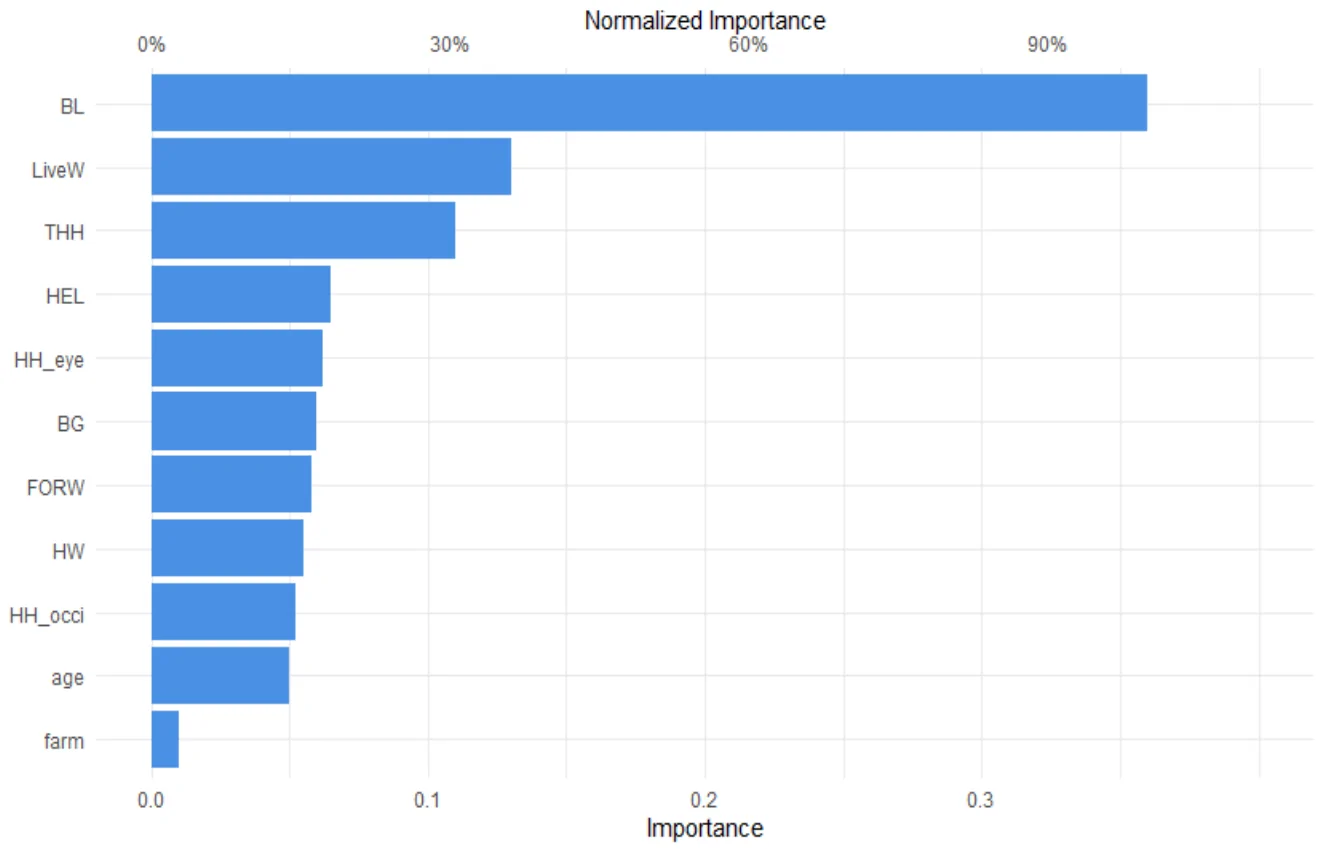
Relative importance of predictors in the ANN model.

The % column graph of the variables that are effective in the prediction is given in Figure 6.

### 3.4. Random Forest (RF) Algorithm Results

**Figure 7:**
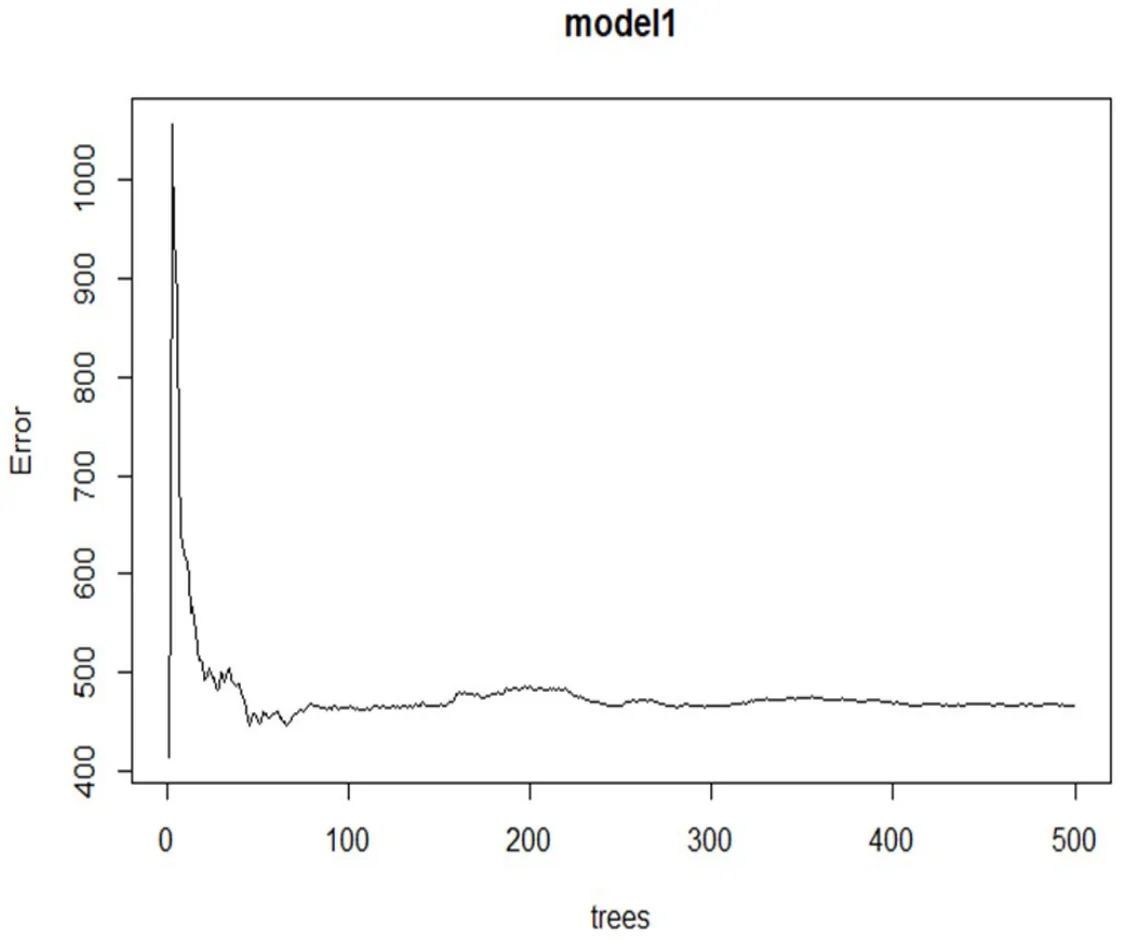
RF Algorithm error rate of the model.

Figure 7 illustrates the changes in model error rate as a function of the number of trees (ntree) in the Random Forest ensemble, demonstrating the convergence of the error rate.

**Figure 8:**
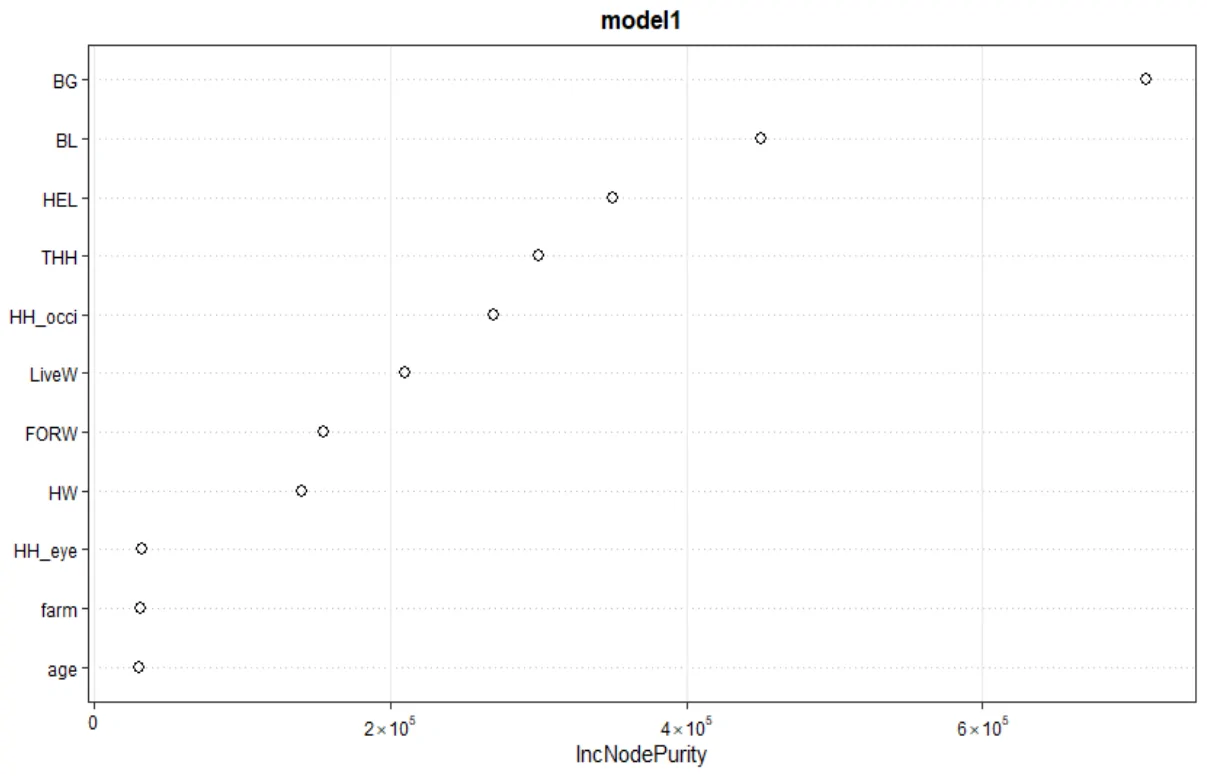
Significance graph of the variables of the RF model.

The RF method was employed to build the model, using body weight as the dependent variable. The RF model incorporates various plant linear characteristics as predictors, including age, farm, BL, THH, BG, HEL, FORW, HH eye, HH occi, LiveW and HW. The random forest algorithm used 500 trees. Body girth (BG) is the most significant factor influencing the model, followed by BL and HEL (as shown in Table 5 and Figure 8).

It is also possible to estimate body weight (BW) by varying the values of the characteristic features that represent the independent variables in the equation generated by the Random Forest (RF) technique. To illustrate this, consider the following calculation: Age = 2, BL = 150, THH = 40, BG = 95, HEL = 40, FORW = 13, HH eye = 19, HH occi = 31, HL = 55.5, LiveW = 0.7, HW = 0.05, farm = “Farm1”, resulting in BW = 142.914 g.

**Figure 9:**
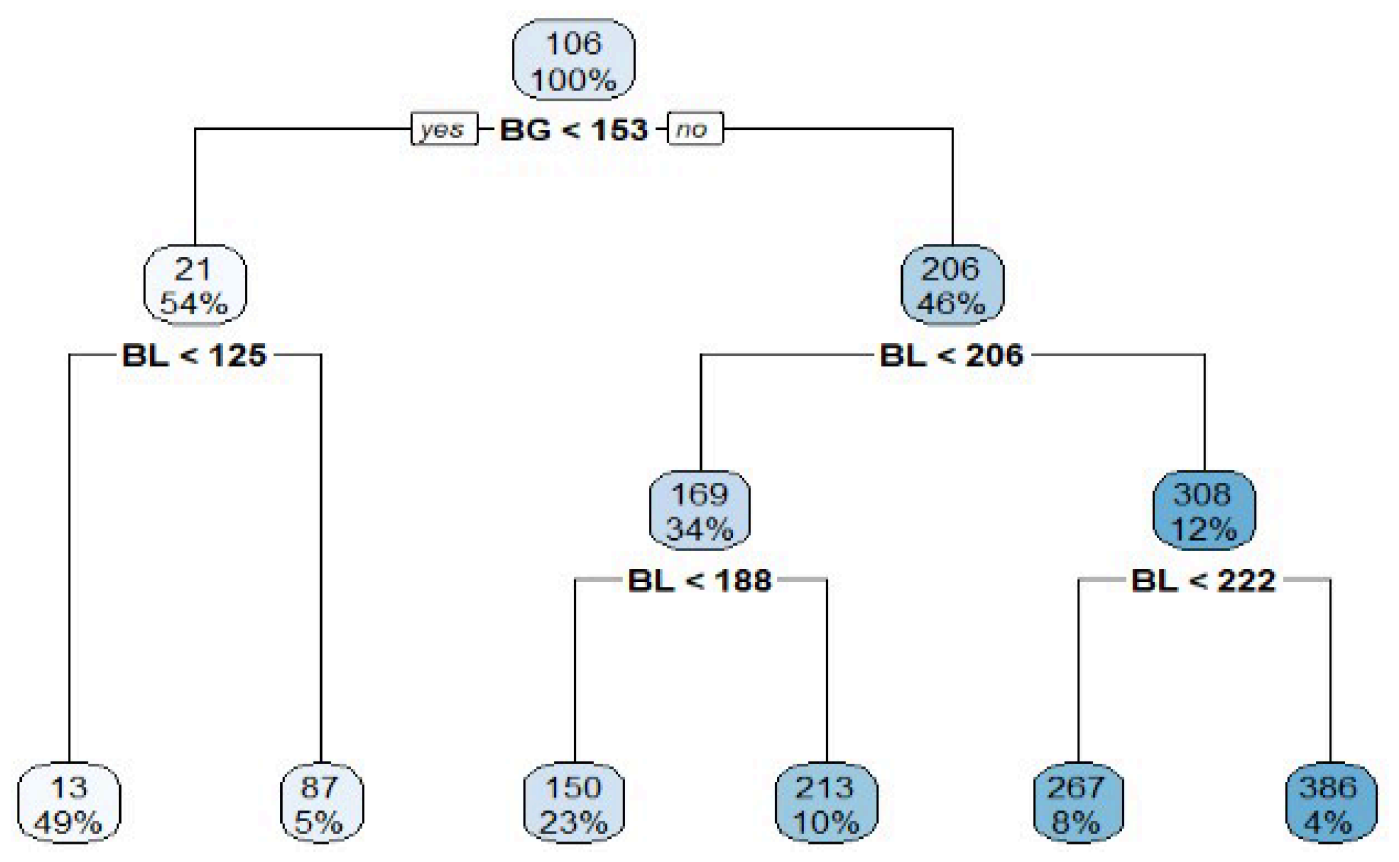
RF algorithm regression tree for BW.

The regression tree depicting the morphological features affecting body weight (BW) in the Random Forest (RF) algorithm is shown in Figure 9.

Examining Figure 9, the following explanations can be derived from the RF algorithm (n = 264 nodes).

(node), split, n, deviance, yval, * denotes terminal node

(1) root 264 3033599.000 105.61290

(2) BG < 153 143 87925.450 20.65524

(4) BL < 125 129 15337.570 13.46512 *

(5) BL ≥ 125 14 4468.449 86.90714 *

(3) BG ≥ 153 121 693720.600 206.01740

(6) BL < 205.5 89 113081.700 169.29100

(12) BL < 187.5 62 33657.400 150.33710 *

(13) BL ≥ 187.5 27 6004.074 212.81480 *

(7) BL ≥ 205.5 32 126717.300 308.16250

(14) BL < 221.5 21 14736.820 267.27140 *

(15) BL ≥ 221.5 11 9831.602 386.22730 *

As shown in Figure 9, examination of the RF tree structure indicated that BG was the primary splitting variable, suggesting its dominant influence on the response variable. Subsequent splits were mainly determined by BL, demonstrating its strong predictive contribution. Higher BL values generally corresponded to substantially greater predicted response values. The highest prediction (BW = 386.23) was observed in the subgroup where BG ≥ 153 and BL ≥ 221.5. These findings indicate that both BG and BL play critical roles in explaining variability in the target trait.

### 3.5. Results of MARS Algorithm

The desirable predictive quality of the MARS equation produced was obtained by ensuring the smallest GCV (117). The results of the importance test of the bound coefficients of the prediction equation produced by the MARS algorithm are presented in Table 6.

In Table 6, according to MARS algorithm results, for the fish with BL > 145, the effect of the second term and corresponding positive coefficient (1.73) on body weight was found positively; the effect of the third term and corresponding negative coefficient (−0.678) on body weight was found adversely in BL < 145. If liver weight was more than 12.2 g, the influence of MARS term number 4 on body weight was also positive, inferring that max(0, LiveW-12.2); whereas liver weight was less than 12.2 g, the influence of MARS term number 5 on body weight was also negative, inferring that max(0, 12.2-LiveW). The sixth MARS term, max(0, BL-145)*max(0, BG-190), and corresponding positive coefficient (0.0281) had a positive effect on body weight. However, the seventh MARS term, max(0, BL-145)*max(0, 190-BG), and corresponding negative coefficient (−0.0244) had a negative effect on body weight. When the eighth and ninth terms, max(0, BG*max(0, LiveW-12.2) and max(0, BL-145)*max(0, 190-BG)*max(0, FORW-22) as well as corresponding coefficients (−1.7 and 0.00323) were taken into consideration, an increment in body weight would be expected for the fish with BL > 145 mm, BG < 190 mm, and FORW > 22 mm, whereas the negative effect of the eighth term, max(0, BG*max(0, LiveW-12.2), on body weight was masked regardless of its coefficient. In the 10th term, when the body length (BL) is more than 145 mm, the body girth (BG) is less than 190 mm, and the forehead width (FORW) is less than 22 mm, the fish’s body weight will increase by 0.0272 g. In the 11th term, when the body length (BL) is greater than 107 mm and the liver weight (LiveW) is less than 12.2, the fish’s body weight will increase by 0.0444 g. In the 12th term of the MARS model, when the body length (BL) is less than 107 mm and the liver weight (LiveW) is less than 12.2, the fish’s body weight will increase by 0.0235 g.

The following is the prediction equation for the MARS results:

BW = 131 − 0.678 * max(0, 145 − BL) + 1.73 * max(0, BL − 145) − 6.91 * max(0, 12.2-LiveW) + 382 * max(0, LiveW-12.2) − 1.7 * BG * max(0, LiveW-12.2) + 0.0281 * max(0, BL-145) * max(0, BG190) − 0.0244 * max(0, BL-145) * max(0, 190-BG) + 0.0235 * max(0, 107 − BL) * max(0, 12.2-LiveW) + 0.0444 * max(0, BL-107) * max(0, 12.2-LiveW) + 0.00323 * max(0, BL-145) * max(0, 190-BG) * max(0, FORW-22) + 0.0272 * max(0, BL-145) * max(0, 190-BG) * max(0, 22-FORW)

**Figure 10:**
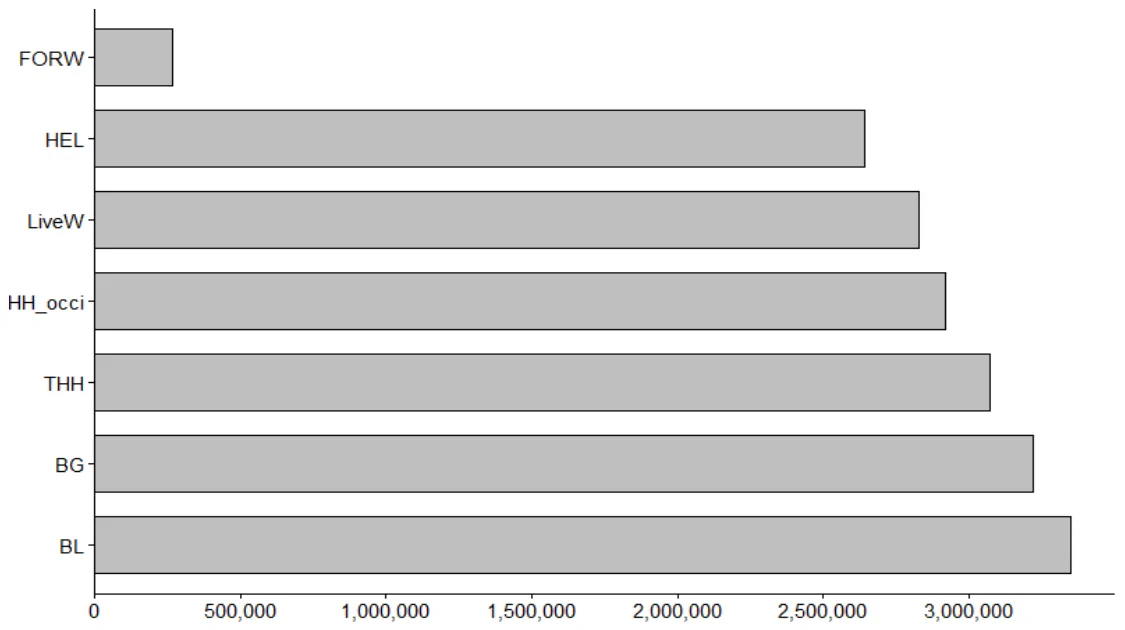
Graph of relative importance for body weight.

The relative importance of the variables predicting body weight as a result of the MARS algorithm is given in Figure 10.

**Figure 11:**
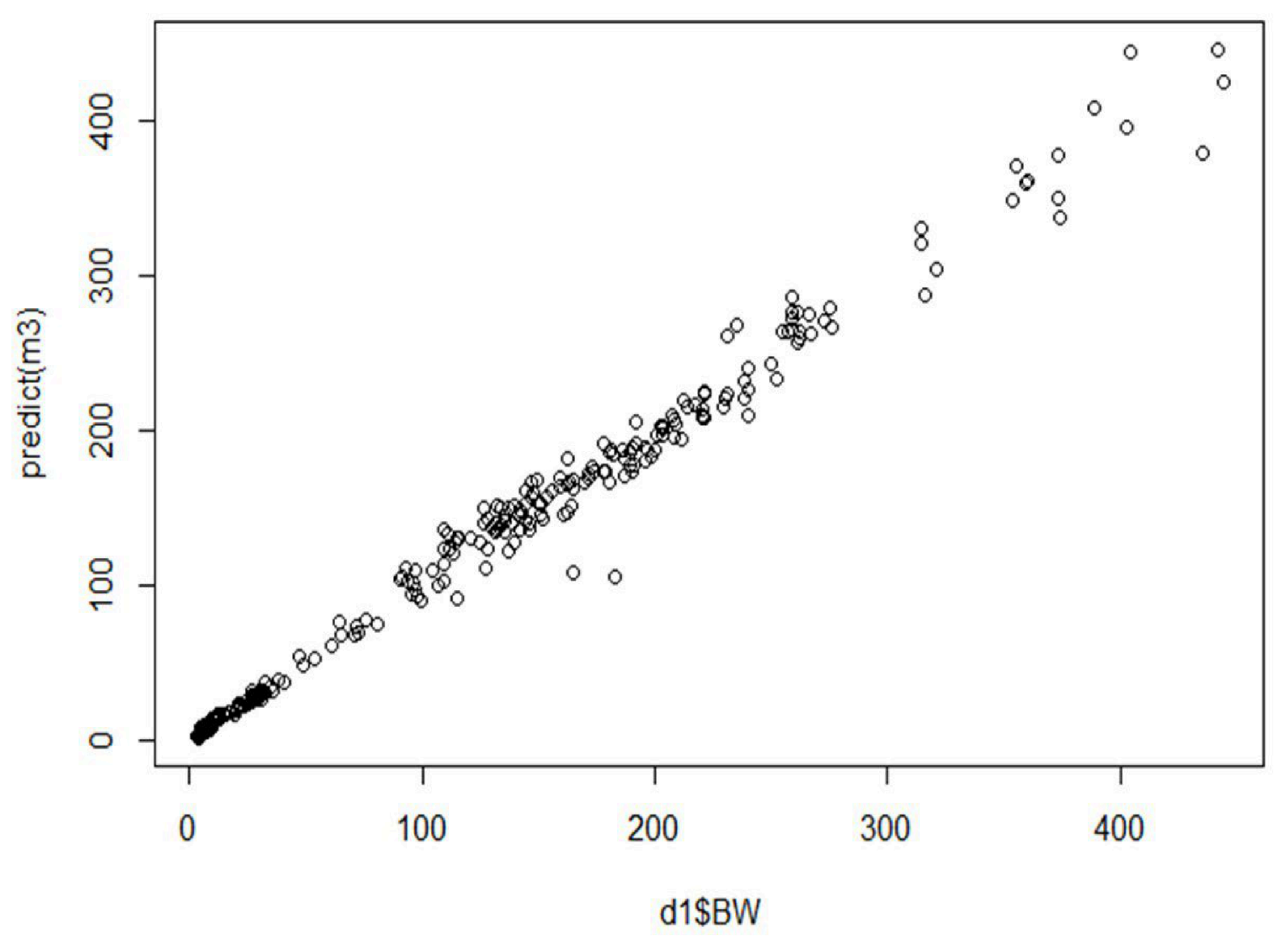
Observed and expected values of the dependent variable (BW).

As shown in Figure 10, the most important variable affecting body weight in fish is body length (BL). BG and THH are the other important variables that follow in sequence. Figure 11 displays the graph of the observed values and the estimated values produced by the MARS algorithm.

Fish with the following traits are assumed to exist: farm = “Farm1”, age = 2, BL = 150, THH = 40, BG = 95, HEL = 40, FORW = 13, HH eye = 19, HH occi = 31, HL = 55.5, LiveW = 0.7, and HW = 0.05. Fish with these traits were expected to weigh 187.312 g in total.

The goodness-of-fit criterion results for all methods are displayed in Table 7. Each algorithm produced quite accurate BW forecasts. In terms of expected accuracy, MARS was found to be the best method, followed by ANN, RF, CART and then CHAID.

The correlation coefficient between the measured and predicted body weights of fish and other body measurements for the CART tree-based algorithm was found to be 0.977 (*p* < 0.01). The coefficient of determination as a goodness-of-fit criterion was 0.954 (R^2^) for the tree-based algorithm. In addition, the SD ratio, the ratio of residuals’ standard deviation to the standard deviation of the observed values, was estimated at 0.214.

RF model explains 95.64% of the variation in the dependent variable, with RMSE = 17.301, MAPE = 11.249, and Bias = 1.5.

For the MARS model with 12 retained terms, the coefficient of determination was R^2^ = 0.989, whereas the cross-validated coefficient of determination (CVR^2^) was 0.966. The difference between R^2^ and CVR^2^ was 0.023, indicating that the predictive performance estimated during model fitting was broadly consistent with the cross-validated performance. However, these values were interpreted as evidence of predictive stability rather than as definitive evidence that overfitting was absent. The standard deviation ratio was 0.103, with an RMSE of 10.801 and a MAPE of 10.757, indicating a high level of predictive accuracy for the MARS model. The standard deviation ratio for ensuring a good fit should be less than 0.40, and for an excellent fit, the standard deviation ratio should be less than 0.10 [54,55,56,57].

The CHAID algorithm also showed strong predictive performance, with a correlation coefficient of 0.973 and an R^2^ value of 0.946, indicating that the model explained approximately 94.6% of the variation in body weight. However, its RMSE and MAPE values were higher than those of the ANN, RF, and MARS models.

The ANN model demonstrated high predictive accuracy with an r value of 0.985 and an R^2^ value of 0.970. In addition, the ANN algorithm produced lower error statistics (RAE, RMSE, and MAD) compared with the CHAID and CART models, indicating better prediction performance.

Although relatively high (R^2^) values were obtained for several models, the error-based metrics indicated differences in their practical prediction accuracy. In particular, the CART model showed a relatively high (R^2^), but its MAPE was approximately 49.6%, indicating substantial relative prediction errors for a considerable proportion of observations. Similarly, the ANN model achieved a relatively high (R^2^), whereas its MAPE of approximately 28.4% indicated that prediction errors remained appreciable when expressed relative to the observed body weights. The MARS model showed the most favorable combination of goodness-of-fit and error-based performance measures among the evaluated models, with high explanatory power and comparatively lower prediction error. Therefore, model performance was assessed using (R^2^) together with RMSE and MAPE rather than relying on a single goodness-of-fit statistic. The results indicate that the algorithms differed not only in their ability to explain variation in body weight but also in the magnitude of their prediction errors.

## 4. Discussion

Fish is a major source of animal protein for the improvement and sustenance of human health [58]. Morphological traits are widely used in the evaluation of body size [59]. Knowledge of body weight and morphometric traits of fish is essential for informed decision-making on farms [60]. The optimization of the production process and cost reduction is synonymous with accurate fish weight prediction, thereby promoting breeding efficiency, sustainability and ecological preservation. Phenotypic relationship permits proper comprehension of the rate and pattern of growth in fish. As body size increased in the present study, there was a corresponding increase in the body linear measurements. The high and positive correlations between body weight and biometric measurements of fish indicate the possibility of estimating one variable from the other. It is possible that genes influencing these traits are to a great extent similar. This information, when validated genotypically, may also aid the genetic improvement of the traits. The range of correlation coefficients (0.83–0.99) obtained in the present study is similar to the values ranging from 0.83 to 0.98 [3] but lower than the values of 0.96–0.99 reported by Karakatsouli et al. (2024) [4]. These differences, however, may be due to genetics, management, environment and the types of traits investigated.

From the five models used in the current study, which helped to eliminate multicollinearity and increase the reliability of the estimation, BL, BG, LiveW, THH, HEL and HH were identified as traits of primary importance in predicting body weight either singly or in combination. The inclusion of body length as an important trait in the prediction of body weight in fish is not surprising, as it has earlier been reported as a veritable trait of interest [1,3,22]. When the five models were compared, the MARS algorithm was found to be the most effective compared to CHAID, CART, ANN and RF. The most associated variable in the MARS equation was BL. This indicates that BL has the greatest contribution to the body weight of the fish. In a related study, relative liver size was found as one of the influencing variables in predicting sablefish maturity [61].

Comparisons of the present MARS findings with previous studies conducted on fish species were limited because of the relatively small number of available studies in the literature. Nevertheless, the MARS algorithm has been successfully applied in other animal species for body weight prediction with high predictive accuracy. Researchers [22] reported that the MARS algorithm produced better body weight predictions in Kıvırcık lambs, where chest width, croup height, and chest circumference were identified as the most important linear body measurements. In that study, the respective R^2^ and adjusted R^2^ values for MARS, CART, CHAID, and RF were reported as 0.792 and 0.768; 0.722 and 0.721; 0.699 and 0.633; and 0.662 and 0.637, respectively.

A similar trend was observed by Mathapo et al. (2025) [62], who found BL to be the best predictor of body weight in male goats using the MARS algorithm. Likewise, [7] reported that body weight in sheep was predicted more accurately from heart girth using the MARS algorithm compared with the CART model. These findings support the effectiveness of the MARS approach in modeling nonlinear relationships between morphometric traits and body weight across animal species.

The predictive performance obtained in the present study can also be considered in relation to recent computer-vision-based approaches for fish weight estimation. Fernandes et al. (2020) [15] developed a deep-learning-based computer vision system for Nile tilapia and used automatically extracted body measurements, including body area, length, height, and eccentricity, to predict body weight and carcass traits. Using images from 1653 fish, their best predictive model included body area and its squared term and explained approximately 96% of the variation in body weight (R^2^ = 0.96). This level of predictive performance demonstrates the potential of image-derived phenotypic measurements as non-invasive indicators of fish body weight. The present study used manually obtained morphometric measurements rather than image-derived traits and evaluated five data-mining algorithms, with MARS providing the best predictive performance within the present dataset and validation design. Although the prediction performance of the two studies should not be compared directly because they involved different fish species, sample sizes, predictor types, and modeling frameworks, both studies indicate that external measures of body size contain substantial information for estimating fish body weight.

The findings of the present study are also consistent with previous applications of MARS in fish research. Koyun and Çelik investigated the relationships between body weight, fish characteristics, and ectoparasite-related variables in Mesopotamian spiny eels (*Mastacembelus mastacembelus*) using MARS and CHAID. In their study, MARS models produced high coefficients of determination, with R^2^ values of 0.973 and 0.989 and RMSE values of 20.823 and 13.598, respectively, depending on the modeled parasite-related response. These findings support the ability of MARS to represent complex relationships involving fish-related biological variables. The relatively strong performance of MARS in both studies may be related to its flexible basis-function structure, which allows nonlinear effects and interactions among predictors to be represented without requiring a single global linear relationship. However, the results should not be interpreted as evidence that MARS will universally outperform other algorithms.

In the present study, 330 individual fish were available, with 110 fish sampled from each of three farms, and each fish contributed one independent observation. The models were evaluated using the same training and testing observations, allowing a direct comparison of the five algorithms under the same data conditions. In addition, previous fish-related data-mining studies have successfully applied predictive modeling to datasets smaller than the present dataset. For example, [16] investigated fish-related biological variables using MARS and CHAID in 122 Mesopotamian spiny eels. Their study illustrates that data-mining methods have also been applied to relatively small fish datasets, although the sample size, predictor structure, response variables, and modeling objectives differed from those of the present study. We therefore use this study only as contextual evidence that fish data-mining applications are not necessarily restricted to very large datasets, rather than as evidence that a sample of 122 or 330 fish is universally sufficient.

Further evidence that the predictive performance of machine-learning algorithms may vary according to the dataset and modeling objective was provided by Benzer et al. (2025) [18], who compared traditional growth models with several machine-learning approaches for Eastern mosquitofish (*Gambusia holbrooki*). Their study included 194 fish collected from the Gediz River and evaluated linear regression, multilayer perceptron, radial basis function network, radial basis function regression, and Sequential Minimal Optimization (SMO) regression. The authors reported that the traditional von Bertalanffy model showed a slightly lower MAPE for length prediction (2.734), whereas different machine-learning algorithms performed differently depending on the response variable; the multilayer perceptron provided favorable results for length prediction, while SMO regression performed better for weight prediction. These findings are relevant to the present study because they demonstrate that the relative performance of machine-learning algorithms is context-dependent and may change according to the species, predictor variables, response variable, and modeling objective. In the present study, MARS showed the most favorable predictive performance among the five evaluated algorithms, whereas the results of [18] indicate that other algorithms may perform better under different biological and data structures. Therefore, the present findings should be interpreted as evidence of the comparative performance of MARS within the current hybrid silver carp dataset rather than as evidence that MARS is universally superior for fish body-weight prediction.

A particularly relevant comparison can be made with the study of [19], which estimated the body weight of *Sparus aurata* using total length, standard length, and fork length as predictor variables. Their dataset consisted of 321 fish (164 females and 157 males), which is comparable in size to the 330 fish evaluated in the present study. The authors compared a multilayer perceptron (MLP), M5P nonlinear regression, and linear regression models. For the total dataset, correlation coefficients were 0.9663, 0.9735, and 0.9473 for MLP, M5P, and linear regression, respectively, while the corresponding MAE values were 2.7074, 2.157, and 3.11. These findings demonstrate that nonlinear and machine-learning approaches can provide accurate body-weight prediction from external morphometric measurements in fish. The study is particularly relevant to the present work because both studies used morphometric characteristics as predictors and involved a similar number of fish.

When evaluating the findings, it is important to take into account a number of the study’s shortcomings. First, 330 hybrid silver carp from just three fish farms in the same area made up the sample. The sample size and geographic coverage may restrict the findings’ applicability to other farms, production systems, environmental circumstances, and geographical areas, even though the dataset contained fish from a variety of age classes and body-size groups. Second, there was variation in body size and age within the dataset. Because the correlations between morphometric parameters and body weight might differ between developmental stages and size ranges, this variability is important for body-weight prediction. This variation may lead to variations in prediction performance among subgroups, even though it may also make the models more applicable within the sampled population. Third, because the morphometric predictors reflect various aspects of the same fish, there was a substantial correlation between them. Such correlations may affect variable importance, model structure, and the stability or interpretation of individual predictor effects, although their influence may differ among the algorithms evaluated. The addition of post-mortem factors, including liver and heart weight, is another drawback. These factors may provide additional predictive information but cannot be obtained from a live fish without invasive or post-mortem procedures. Consequently, models incorporating these variables have limited applicability for non-destructive body-weight estimation in routine aquaculture practice. Finally, although the dataset was randomly divided into training and testing subsets and the same testing observations were used for comparison of all algorithms, the study did not include independent external validation using fish from farms, production periods, or geographical regions that were not represented in model development. In addition, random splitting at the individual-fish level may allow the training and testing subsets to contain fish originating from the same farms and production conditions. Therefore, the reported test-set performance primarily reflects the ability of the models to generalize to new individual fish sampled under conditions represented in the original dataset, rather than demonstrating generalization to entirely new farms or production environments.

Regarding practical application, the structural complexity and predictor requirements of the fitted models dictate their operational context. Algorithms such as MARS identified significant interaction terms between external traits and internal organ parameters (e.g., the interaction between BG and LiveW). While models retaining LiveW or HW yield superior explanatory power for total body weight determination, making them highly valuable for processing plant evaluations, yield accounting, and biological growth research; their application is inherently post-mortem. For operational scenarios where non-lethal estimation of body weight is required prior to processing, future model variants restricted strictly to external physical measurements (BL, BG) should be prioritized.

## 5. Conclusions

The present study demonstrated that machine learning algorithms can successfully predict fish body weight using morphometric traits, age, and farm-related information. Strong positive relationships were observed among the measured morphological traits, indicating that increases in body dimensions were generally associated with increases in body weight.

Across the evaluated algorithms, body length, body girth, the highest height, and head-related measurements were identified as the most important predictors affecting fish body weight. These findings suggested that morphometric traits can serve as reliable indicators for estimating body weight in hybrid silver carp. Among the tested machine learning approaches, the MARS algorithm showed the best predictive performance for body weight estimation. Therefore, MARS may be considered an effective and reliable tool for modeling complex nonlinear relationships among fish morphometric characteristics.

However, the findings should be interpreted within the scope of the present dataset and validation design. The fish were obtained from three farms within the same geographical region, and the random train–test partition does not constitute independent external validation across farms or production systems. Therefore, the predictive performance of MARS observed in this study should not be considered evidence of broad applicability to other fish populations or production conditions. Independent external validation using fish from additional farms, geographical regions, and production environments is warranted before the MARS model can be considered a broadly applicable tool for practical body-weight prediction.

## Figures and Tables

**Table 1:** Descriptive statistics of body morphologic characteristics of fish.

	Farm	N	$\bar{X}$	$s_{\bar{x}}$	s	Min.	Max.
Age	Farm1	110	1.927	0.063	0.664	1	2
	Farm2	110	1.952	0.061	0.644	1	2
	Farm3	110	1.969	0.062	0.652	1	2
BL	Farm1	110	153.864	5.355	56.162	65	258
	Farm2	110	131.527	6.053	63.482	52	236
	Farm3	110	121.555	4.848	50.843	57	220
THH	Farm1	110	61.027	2.105	22.077	26	100
	Farm2	110	47.436	2.372	24.878	18	89
	Farm3	110	46.064	2.090	21.922	18	88
BG	Farm1	110	153.064	5.609	58.825	52	246
	Farm2	110	123.118	5.787	60.694	50	221
	Farm3	110	116.127	4.896	51.351	49	215
HEL	Farm1	110	66.855	2.570	26.955	29	110
	Farm2	110	50.264	2.364	24.797	18	91
	Farm3	110	45.909	1.994	20.917	20	88
FORW	Farm1	110	24.673	1.151	12.071	10	48
	Farm2	110	17.655	1.007	10.562	4	36
	Farm3	110	16.218	0.893	9.367	5	39
HH eye	Farm1	110	31.545	1.191	12.492	15	56
	Farm2	110	23.209	1.133	11.882	7	43
	Farm3	110	21.791	0.997	10.453	8	48
HH occi	Farm1	110	51.282	1.956	20.514	20	85
	Farm2	110	41.009	2.059	21.594	15	76
	Farm3	110	39.755	1.811	18.991	16	82
LiveW	Farm1	110	5.225	0.460	4.82	0.4	21.5
	Farm2	110	3.469	0.307	3.22	0.1	11.5
	Farm3	110	1.926	0.171	1.789	0.1	5.3
HW	Farm1	110	0.402	0.034	0.359	0.05	1.3
	Farm2	110	0.334	0.030	0.318	0.01	1
	Farm3	110	0.232	0.025	0.262	0.01	1
BW	Farm1	110	144.422	12.113	127.04	4.7	444
	Farm2	110	106.171	9.532	99.976	3.6	373
	Farm3	110	70.497	6.431	67.449	2.8	259

BW: Body weight, LiveW: liver weight, HW: heart weight, HEL: head length, FORW: forehead width, HH eye: head height through the eye line, HH occi: head height through the occiput, BL: body length, THH: the highest height, BG: body girth.

**Table 2:** Correlation coefficients between the body morphological characteristics of fish.

Variable		BL	THH	BG	HEL	FORW	HH Eye	HH Occi	LiveW	HW	BW
BL	r	1	0.987 **	0.989 **	0.957 **	0.951 **	0.949 **	0.985 **	0.880 **	0.881 **	0.944 **
	*p*		0.001	0.001	0.001	0.001	0.001	0.001	0.001	0.001	0.001
THH	r	0.987 **	1	0.993 **	0.970 **	0.968 **	0.965 **	0.991 **	0.871 **	0.885 **	0.932 **
	*p*	0.001		0.001	0.001	0.001	0.001	0.001	0.001	0.001	0.001
BG	r	0.989 **	0.993 **	1	0.974 **	0.969 **	0.965 **	0.991 **	0.878 **	0.888 **	0.938 **
	*p*	0.001	0.001		0.001	0.001	0.001	0.001	0.001	0.001	0.001
HEL	r	0.957 **	0.970 **	0.974 **	1	0.985 **	0.987 **	0.980 **	0.874 **	0.891 **	0.917 **
	*p*	0.001	0.001	0.001		0.001	0.001	0.001	0.001	0.001	0.001
FORW	r	0.951 **	0.968 **	0.969 **	0.985 **	1	0.986 **	0.976 **	0.870 **	0.907 **	0.923 **
	*p*	0.001	0.001	0.001	0.001		0.001	0.001	0.001	0.001	0.001
HH eye	r	0.949 **	0.965 **	0.965 **	0.987 **	0.986 **	1	0.975 **	0.866 **	0.893 **	0.906 **
	*p*	0.001	0.001	0.001	0.001	0.001		0.001	0.001	0.001	0.001
HH occi	r	0.985 **	0.991 **	0.991 **	0.980 **	0.976 **	0.975 **	1	0.870 **	0.892 **	0.926 **
	*p*	0.001	0.001	0.001	0.001	0.001	0.001		0.001	0.001	0.001
LiveW	r	0.880 **	0.871 **	0.878 **	0.874 **	0.870 **	0.866 **	0.870 **	1	0.825 **	0.906 **
	*p*	0.001	0.001	0.001	0.001	0.001	0.001	0.001		0.001	0.001
HW	r	0.881 **	0.885 **	0.888 **	0.891 **	0.907 **	0.893 **	0.892 **	0.825 **	1	0.892 **
	*p*	0.001	0.001	0.001	0.001	0.001	0.001	0.001	0.001		0.001
BW	r	0.944 **	0.932 **	0.938 **	0.917 **	0.923 **	0.906 **	0.926 **	0.906 **	0.892 **	1
	p	0.001	0.001	0.001	0.001	0.001	0.001	0.001	0.001	0.001	

BW: Body weight, LiveW: liver weight, HW: heart weight, HEL: head length, FORW: forehead width, HH eye: head height through the eye line, HH occi: head height through the occiput, BL: body length, THH: the highest height, BG: body girth. **: *p* < 0.01).

**Table 3:** Parameter estimates of the BW optimal ANN model.

		H(1:1)	H(1:2)	H(1:3)	H(1:4)	H(1:5)	H(1:6)	H(1:7)	BW
Input Layer	(Bias)	−0.402	−0.038	−0.234	0.352	0.103	0.633	0.414	
	[farm = 1.00]	0.348	−0.398	0.341	−0.027	−0.289	0.458	0.287	
	[farm = 2.00]	0.082	−0.226	0.199	0.156	0.223	0.414	0.254	
	[farm = 3.00]	0.404	−0.166	0.266	0.405	−0.226	0.460	0.233	
	Age	−0.509	0.412	−0.528	0.401	−0.051	−0.012	−0.052	
	BL	−0.026	0.178	−0.275	0.253	−0.319	−0.305	−0.717	
	THH	0.245	0.109	−0.456	−0.113	0.110	−0.074	−0.185	
	BG	0.120	−0.266	0.356	0.327	0.258	−0.543	0.201	
	HEL	−0.400	0.007	−0.461	0.474	−0.276	−0.080	0.430	
	FORW	−0.322	−0.021	0.367	0.091	0.070	−0.392	0.130	
	HH eye	0.152	0.471	0.363	0.291	−0.156	−0.287	0.043	
	HH occi	−0.378	−0.235	0.405	−0.084	−0.249	0.030	−0.081	
	LiveW	0.204	0.001	0.127	0.262	0.305	0.190	−0.471	
	HW	0.087	−0.156	−0.426	0.357	−0.295	0.302	−0.018	
Hidden Layer 1	(Bias)								0.859
	H(1:1)								−0.181
	H(1:2)								−0.334
	H(1:3)								−0.346
	H(1:4)								−0.210
	H(1:5)								−0.181
	H(1:6)								−0.950
	H(1:7)								−1.006

**Table 4:** Independent variable importance.

Variables	Importance	Normalized Importance
Farm	0.007	1.8%
Age	0.048	13.4%
BL	0.358	100.0%
THH	0.109	30.5%
BG	0.059	16.5%
HEL	0.067	18.6%
FORW	0.057	15.9%
HH eye	0.062	17.4%
HH occi	0.049	13.8%
LiveW	0.131	36.5%
HW	0.054	15.1%

**Table 5:** Predictor importance in RF.

Variable	Node Purity
Age	4690.557
Farm	4984.670
BL	451,489.686
THH	301,336.723
BG	712,991.036
HEL	352,397.919
FORW	155,120.147
HH eye	29,677.968
HH occi	266,684.546
LiveW	206,756.448
HW	138,141.926

**Table 6:** Results of the MARS algorithm in the prediction of body weight of fish.

Terms	Basis Function	Coefficients	Std. Error	*t* Value	Pr (>|t|)
1	(Intercept)	1.315 × 10^2^	8.163 × 10^0^	16.105	<2 × 10^−16^ ***
2	max(0, BL-145)	1.731 × 10^0^	1.645 × 10^−1^	10.524	<2 × 10^−16^ ***
3	max(0, 145-BL)	−6.779 × 10^−1^	2.782 × 10−1	−2.437	0.0154 *
4	max(0, LiveW-12.2)	3.822 × 10^2^	3.262 × 10^1^	11.717	<2 × 10^−16^ ***
5	max(0, 12.2-LiveW)	−6.908 × 10^0^	1.563 × 10^0^	−4.419	1.36 × 10^−5^ ***
6	max(0, BL-145)*max(0, BG-190)	2.813 × 10^−2^	2.138 × 10^−3^	13.158	<2 × 10^−16^ ***
7	max(0, BL-145)*max(0, 190-BG)	−2.437 × 10^−2^	3.595 × 10^−3^	−6.780	5.86 × 10^−11^ ***
8	max(0, BG*max(0, LiveW-12.2)	−1.700 × 10^0^	1.358 × 10^−1^	−12.520	<2 × 10^−16^ ***
9	max(0, BL-145)*max(0, 190-BG)*max(0, FORW-22)	3.234 × 10^−3^	7.347 × 10^−4^	4.401	1.47 × 10^−5^ ***
10	max(0, BL-145)*max(0, 190-BG)*max(0, 22-FORW)	2.719 × 10^−2^	3.749 × 10^−3^	7.253	3.13 × 10^−12^ ***
11	max(0, BL-107)*max(0, 12.2-LiveW)	4.443 × 10^−2^	1.844 × 10^−2^	2.409	0.0166 *
12	max(0, 107-BL)*max(0, 12.2-LiveW)	2.346 × 10^−2^	2.190 × 10^−2^	1.071	0.2849

*: (*p* < 0.05), ***: (*p* < 0.001).

**Table 7:** Predictive performance of CHAID, CART, ANN, RF and MARS algorithms.

Statistics	CHAID	CART	ANN	RF	MARS	Acceptable Range	The Most Suitable Method
r	0.973	0.977	0.985	0.978	0.995	The highest value	MARS
R^2^	0.946	0.954	0.970	0.956	0.989	The highest value	MARS
Adj. R^2^	0.945	0.953	0.968	0.954	0.988	The highest value	MARS
RAE	0.163	0.150	0.122	0.141	0.072	The lowest value	MARS
RMSE	24.388	22.510	18.353	17.301	10.801	The lowest value	MARS
MAPE	17.237	49.631	28.358	11.249	10.757	The lowest value	MARS
MAD	13.141	14.552	9.317	9.634	6.234	The lowest value	MARS
SD_ratio_	0.232	0.214	0.175	0.271	0.103	The lowest value	MARS
AIC	2118.105	2065.213	1930.464	2138.238	1595	The lowest value	MARS
AICc	2118.291	2065.398	1930.649	2138.976	1596	The lowest value	MARS

MARS—multivariate adaptive regression spline; CHAID—chi-square automatic interaction detection; CART—classification and regression tree; ANN—artificial neural network; AIC—Akaike information criterion; AICc—corrected Akaike information criterion; RMSE—root-mean-square error; SDratio—standard deviation ratio; MAPE—mean absolute percentage error; R^2^—coefficient of determination.

## Data Availability

The original contributions presented in this study are included in the article. Further inquiries can be directed to the corresponding authors.

## Abbreviations

The following abbreviations are used in this manuscript:

Abbreviation	Definition
AIC	Akaike Information Criterion
ANN	Artificial Neural Network
BG	Body Girth
BL	Body Length
BW	Body Weight
CART	Classification and Regression Trees
CHAID	Chi-Squared Automatic Interaction Detector
FORW	Forehead Width
HEL	Head Length
HH eye	Head Height through the Eye Line
HH occi	Head Height through the Occiput
HW	Heart Weight
LiveW	Liver Weight
MAE	Mean Absolute Error
MAPE	Mean Absolute Percentage Error
MARS	Multivariate Adaptive Regression Splines
RAE	Relative Approximation Error
RF	Random Forest
RMSE	Root Mean Square Error
THH	The Highest Height
